# Increased Circulating Cathepsin K in Patients with Chronic Heart Failure

**DOI:** 10.1371/journal.pone.0136093

**Published:** 2015-08-24

**Authors:** Guangxian Zhao, Yuzi Li, Lan Cui, Xiang Li, Zhenyi Jin, Xiongyi Han, Ennan Fang, Yihua Gao, Dongmei Zhou, Haiying Jiang, Xueying Jin, Guanghao Piao, Xiangshan Li, Guang Yang, Jiyong Jin, Enbo Zhu, Meina Piao, Limei Piao, Kuichang Yuan, Yanna Lei, Dazhi Ding, Chengzhi Jin, Yongshan Nan, Xianwu Cheng

**Affiliations:** 1 Department of Cardiology, Yanbian University Hospital, Yanji, Jilin, P.R., China; 2 Department of Clinical Examination, Yanbian University Hospital, Yanji, Jilin, P.R., China; 3 Department of Central Laboratory, Yanbian University Hospital, Yanji, Jilin, P.R., China; 4 Department of Physiology and Pathophysiology, Yanbian University Medical College, Yanji, Jilin, P.R., China; 5 Department of Anesthesiology, Yanbian University Hospital, Yanji, Jilin P.R., China; Katholieke Universiteit Leuven, BELGIUM

## Abstract

Cysteinyl cathepsin K (CatK) is one of the most potent mammalian collagenases involved in cardiovascular disease. Here, we investigated the clinical predictive value of serum CatK levels in patients with chronic heart failure (CHF). We examined 134 patients with CHF, measuring their serum CatK, troponin I, high-sensitive C-reactive protein, and pre-operative N-terminal pro-brain natriuretic peptide levels. The patients were divided into two groups: the 44 patients who showed a left ventricular (LV) ejection fraction (LVEF) < 40% (the “lowLVEF” group) and the 90 patients showing LVEF values ≥ 40% (the “highLVEF” group). The lowLVEF patients had significantly higher serum CatK levels compared to the highLVEF patients (58.4 ± 12.2 vs. 44.7 ± 16.4, *P* < 0.001). Overall, a linear regression analysis showed that CatK levels correlated negatively with LVEF (r = −0.4, P < 0.001) and positively with LV end-diastolic dimensions (r = 0.2, *P* < 0.01), LV end-systolic dimensions (r = 0.3, *P* < 0.001), and left atrial diameters (r = 0.3, *P* < 0.01). A multiple logistic regression analysis showed that CatK levels were independent predictors of CHF (odds ratio, 0.90; 95% confidence interval, 0.84–0.95; *P* < 0.01). These data indicate that elevated levels of CatK are closely associated with the presence of CHF and that the measurement of circulating CatK provides a noninvasive method of documenting and monitoring the extent of cardiac remodeling and dysfunction in patients with CHF.

## Introduction

Members of the cathepsin family were original identified as proteases that act the lysosome [1–3]. Recent studies have discovered nontraditional roles for cathepsins in the intracellular and the extracellular space in angiogenesis and cardiovascular disease [4–8]. Among the cysteinyl cathepsins, cathepsin K (CatK), one of the most potent mammalian collagenases, was first identified in inflammatory macrophages and later characterized as the key enzyme in bone resorption by osteoclasts [9–11]. A number of clinical and experimental studies demonstrated that CatK abounds in endothelial cells and vascular smooth muscle cells and inflammatory macrophages of advanced atherosclerotic plaques [12–17]. Consistent with these biochemical observations with vascular cells [18], cardiac myocytes from atrial and ventricular tissues can also secrete CatK that degrades type I collagen and other extracellular matrix (ECM) components of the cardiovascular wall [4,19–21]. Given that genetic and pharmacological interventions targeted toward CatK ameliorate atrial and cardiac fibrosis and dysfunction in animals [7,8,21–23], we hypothesized that circulating CatK levels are associated with cardiac remodeling and dysfunction in patients with chronic heart failure (CHF). We tested this hypothesis in the present study in patients with CHF in order to explore the relationship between circulating CatK and clinical presentations, and we attempted to identify useful noninvasive blood biomarkers that are suggestive of patients with CHF.

## Materials and Methods

### Study population and definition

We recruited 134 consecutive patients with CHF who were admitted to Yanbian University Hospital (Yanji, China) between March 2012 and March 2014 for the in-hospital treatment of decompensation of CHF. All enrolled patients had New York Heart Association (NYHA) functional class II–IV and CHF with a reduced ejection fraction of ischemic (having myocardial infraction history), hypertension (diagnosis of primary hypertension), and idiopathic dilated cardiomyopathic etiologies. These CHF patients were taking standard medical therapeutics with diuretics, inotropic agents (e.g., digoxin), statins, a β-blocker, angiotensin-converting enzyme inhibitors (ACEI) and/or angiotensin type 1 receptor blockers (ARBs). We divided the CHF patients into two groups by their left ventricular (LV) ejection fraction (LVEF) values: the 44 patients showing LVEF < 40% (the “lowLVEF” group) and the 90 patients showing LVEF values ≥ 40% (the “highLVEF” group).

Based on the elevation of cardiac biomarkers (at least one positive biomarker: creatine kinase-MB or troponin T), an electrocardiogram indicative of new ischemia (new ST-T change or new left bundle branch block), and a history of prolonged chest pain, acute myocardial infarction was diagnosed [24]. We defined hypertension as systolic blood pressure > 140 mmHg, diastolic blood pressure > 90 mmHg, and/or having received antihypertensive drugs. Dilated cardiomyopathy was diagnosed on the basis of clinical, electrocardiographic and diagnostic criteria [25,26]. Diabetes mellitus was diagnosed when the patient had a history of any antihyperglycemic medication or a previous diagnosis of diabetes and/or an HbA1c level ≥ 6.5%, a fasting plasma glucose concentration > 126 mg/dL [24].

Patients with prior evidence of end-stage renal disease with maintenance hemodialysis, congenital heart disease, pericarditis, primary valvular disease, hypertrophic cardiomyopathy, acute myocarditis, or secondary cardiac muscle disease caused by any known systemic condition, were excluded. This study protocol was approved by the Ethics Committee of Yanbian University Hospital, and written informed consent was obtained from all patients.

### Laboratory examination

Venous blood samples were obtained for chemical analysis after an overnight fast. Serum CatK levels were evaluated by using enzyme-linked immunosorbent assay (ELISA) kits (Biomedica Gruppe, Biomedica Medizinprodukte, Vienna, Austria) in duplicate. The levels of N-terminal pro-brain natriuretic peptide (NTproBNP), troponin I, low-density lipoprotein (LDL), high-density lipoprotein (HDL), high-sensitive C-reactive protein (hs-CRP), and hemoglobin A1c were studied at the clinical laboratory of Yanbian University Hospital (Clinical Laboratory, Yanji, China). We presented circulating CatK values as ng/mL.

### Echocardiography

We performed echocardiography using a Sonos 2500 ultrasound system (Hewlett-Packard, Andover, MA) equipped with a 2.5- to 3.5-MHz transducer [26]. The LV posterior wall thickness (LVPWT), interventricular septal thickness (IVST), LV end-diastolic dimension (LVDd), LV end-systolic dimension (LVSd), and LVEF were calculated on the M-mode of the long-axis image according to standard criteria recommended by American Society of Echocardiography (ASE). The images were recorded on a DVD recorder and analyzed offline. The left atrial diameter (LAD) was calculated by using standard M-mode measurements, as recommended by the ASE [4].

### Statistical analysis

Continuous variables are expressed as mean 6 SD or as median and interquartile range for nonparametric variables. Categoric variables were compared with the Pearson chi-square test or the Fisher exact test. Comparison of continuous variables between groups was performed with the unpaired Student’s *t-*test or a one-way analysis of variance (ANOVA) followed by a Tukey post hoc test. The levels of hs-CRP, NTproBNP, and troponin I were logarithmically transformed because the data showed a skewed distribution. If the homogeneity of variance assumption was violated, the nonparametric Kruskal-Wallis test was used instead. The correlation analysis was performed using a linear regression analysis. The factors that related at the *P* < 0.1 level were isolated as independent variable candidates for the multiple logistic regression analysis, which was used to evaluate the independent contribution of clinical parameters to CHF. StatFlex (version 6.0; Artech, Osaka, Japan) was used for all statistical analyses. *P-*values < 0.05 were considered significant.

## Results

### Patient characteristics

The patient characteristics are presented in Table 1 and S1 Table. Their mean age was 67.6 ± 14.1 years. According to the NYHA functional classification, the 134 patients were distributed as follows: 17 with NYHA class II CHF, 47 with NYHA class III, and 70 with NYHA class IV. The LVDd and LVDs values derived from echocardiography were 55.9 ± 14.5 mm and 48.7 ± 11.9 mm, respectively, suggesting LV dilation. The IVST and LVPWT values were 9.3 ± 1.5 mm and 12.5 ± 2.1 mm, respectively. The LVEF was reduced to a mean value of 41.9 ± 8.3%. The plasma NTproBNP and CatK levels were 4024 ± 4026 pg/mL and 51.6 ± 16.6 pg/mL, respectively, suggesting elevated neurohumoral factor and CatK.

**Table 1:** Patients’ characteristics.

Age, yrs	67.6 ± 14.1
Female, %	50%
Body mass index, kg/m^2^	23.3 ± 3.9
**NYHA functional class**	
II	12.7
III	35.1
VI	52.2
**Echocardiography**	
LAD, mm	42.3 ± 9.4
IVST, mm	9.3 ± 1.2
LVPWT, mm	12.9 ± 2.0
LVDd, %	55.9 ± 14.5
LVSd, %	48.7 ± 11.9
LVEF, %	41.9 ± 8.3
CI, %	2.8 ± 1.1
**Blood Examination**	
Na^+^, mmol/L	139.9 ± 3.7
LDL, mg/dL	90.7 ± 31.0
HDL, mg/dL	45.5 ± 16.2
Hemoglobin A1c, %	5.9 ± 1.4
Creatinine, mmol/L	75.0 ± 32.2
hs-CRP, mg/dL	4.8 ± 7.2
NTproBNP (pg/mL)	4024 ± 4026
Troponin I (pg/mL)	1.5 ± 4.6
CatK, ng/mL	51.6 ± 16.6

Values are expressed as mean ± SD or percentage (%).

LAD, left atrial dia.; LV, left ventricular; IVST, interventricular septal thickness; LVPWT, LV posterior wall thickness; LVDd, left ventricular end-diastolic dimension; LVSd; LV end-systolic dimension; CI, cardiac index; LDL, low-density lipoprotein; HDL, high-density lipoprotein; hs-CRP, high-sensitivity C-reactivity protein; NTproBNP, N-terminal pro-brain natriuretic peptide; ACEI, angiotensin-converting enzyme inhibitor; ARB, angiotensin type 1 receptor blocker.

### Comparison of each parameter between the two groups

Table 2 shows the clinical characteristics of the two patient groups (lowLVEF group, n = 44; highLVEF group, n = 90). Unlike the gender distributions of the groups (Table 2), there were no significant between-group differences in the patients’ age or BMI values. Patients in the lowLVEF group were significantly more likely to have had cerebrovascular disease, or to have undergone an angioplasty (Table 2 and S2 Table). LowLVEF patients had taken more mineralocorticoid receptor antagonists as well as diuretic and digoxin than had the highLVEF patients.

**Table 2:** Comparison of each parameter between the lowLVEF and highLVEF groups.

	lowLVEF (n = 44)	highLVEF (n = 90)	*P*-value
Age, yrs	63.1 ± 13.2	69.8 ± 10.9	0.09
Female, %	34.1	57.8	0.01
Body mass index, kg/m^2^	23.6 ± 3.6	22.9 ± 4.0	0.60
**Clinical histories**			
Hypertension, %	38.6	31.1	0.12
Diabetes mellitus, %	27.3	22.2	0.52
Current smokers, %	34.1	32.2	0.83
Previous myocardial infarction, %	36.4	34.8	0.86
Previous angioplasty, %	6.8	16.9	< 0.05
Previous bypass surgery, %	0	0	0
Previous cerebrovascular disease, %	2.3	17.8	< 0.05
**Echocardiography**			
LAD, mm	44.9 ± 8.0	41.0 ± 8.3	< 0.01
IVST, mm	8.9 ± 1.3	9.6 ± 1.2	0.12
LVPWT, mm	11.3 ± 1.9	13.7 ± 1.6	0.08
LVDd, %	62.8 ± 11.1	50.9 ± 7.5	< 0.01
LVSd, %	55.4 ± 10.0	43.9 ± 3.6	< 0.01
LVEF, %	32.3 ± 6.5	46.6 ± 3.8	< 0.05
CI, %	2.6 ± 1.2	2.9 ± 0.7	0.33
**Blood Examination**			
Na^+^, mmol/L	139.8 ± 4.4	140.0 ± 3.4	0.74
LDL, mg/dL	90.5 ± 32.5	91.3 ± 28.1	0.81
HDL, mg/dL	44.9 ± 15.4	46.7 ± 17.9	0.77
Hemoglobin, g/dL	12.9 ± 2.2	14.6 ± 2.3	0.09
Serum albumin, g/mL	39.9 ± 5.1	40.5 ± 4.1	0.51
Hemoglobin A1c, %	6.1 ± 1.4	5.8 ± 0.4	0.38
Creatinine, mmol/L	77.4 ± 24.3	73.9 ± 35.4	0.56
hs-CRP, mg/dL	4.3 ± 4.1	5.4 ± 8.4	0.39
NTproBNP (pg/mL)	4269 ± 3757	3599 ± 4225	0.68
Troponin I (pg/mL)	2.1 ± 7.6	1.5 ± 2.3	0.21
CatK, ng/mL	58.4 ± 12.2	44.7 ± 16.4	< 0.001
**Medications**			
ACEIs, %	38.6	37.8	0.92
ARBs, %	47.7	34.4	0.14
β-blockers, %	75.0	71.1	0.64
Statins, %	63.6	64.4	0.64
MR antagonists, %	77.3	56.7	0.02
Diuretics, %	70.5	55.6	<0.05
Digoxin, %	27.3	15.6	<0.05
Insulin, %	11.3	17.8	0.19

Values are expressed as mean ± SD or percentage (%).

LDL, low-density lipoprotein; HDL, high-density lipoprotein; hs-CRP, high-sensitivity C-reactivity protein; NTproBNP, N-terminal Pro-brain Natriuretic Peptide; ACEI, angiotensin-converting enzyme inhibitor; ARB, angiotensin type 1 receptor blocker; MR, mineralocorticoid receptor.

There were no significant differences in IVST or LVPWT between the lowLVEF and highLVEF groups (Table 2). As anticipated, the lowLVEF patients had significantly greater LVDd (62.8 ± 11.1 vs. 50.9 ± 7.5, mm; *P* < 0.01), LVDs (55.4 ± 10.0 vs. 43.9 ± 3.6, mm; *P* < 0.01), and LAD (44.9 ± 8.0 vs. 41.0 ± 8.3, mm; *P* < 0.01) values compared to the highLVEF patients.

### Circulating biomarkers

As shown in Table 2, compared with the highLVEF group, the serum CatK levels were significantly increased in the patients in the lowLVEF group (44.7 ± 16.4 vs. 58.4 ± 12.2, ng/mL; *P* < 0.001), whereas there were no significant differences in blood Na^+^, lipid profile (LDL and HDL), hemoglobin A1c, creatinine clearance rate, hs-CRP, NTproBNP, or triponin I between the groups.

**Fig 1:**
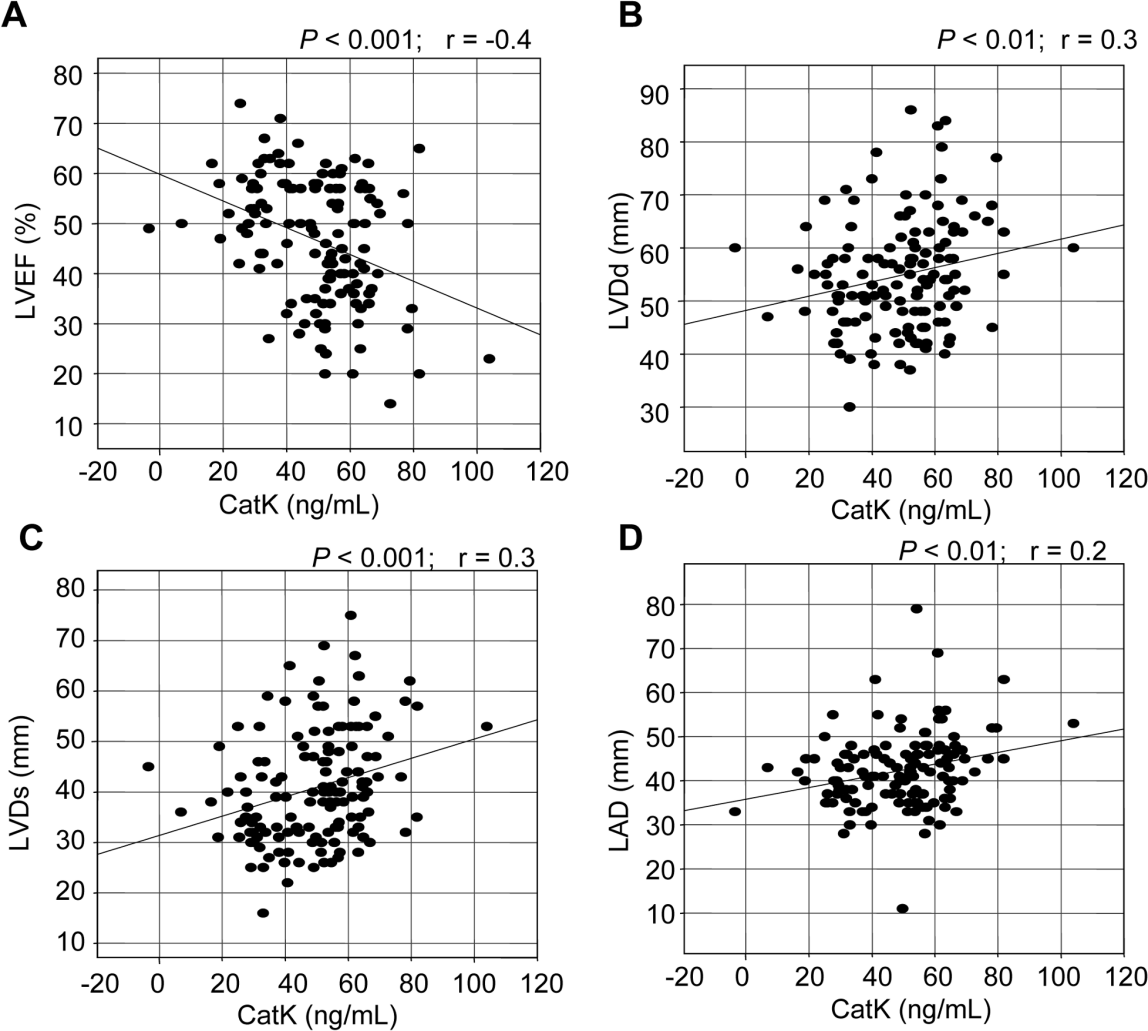
Correlations between serum levels of CatK and left ventricular (LV) ejection fraction (LVEF) as well as LV end-diastolic dimension (LVDd), LV end-systolic dimension (LVDs) and left atrial diameter (LAD). In all patients, there were negative correlations between the levels of CatK and LVEF and positive correlations between the CatK levels and LVDd, LVDs, and LAD.

Our linear regression analysis showed that the CatK levels correlated negatively with the LVEF (*r* = −0.4, *P* < 0.001) and correlated positively with the LVDd (*r* = 0.2, *P* < 0.01) and LVDs (*r* = 0.3, *P* < 0.001) levels (Fig 1 and S1 Fig). There was also a significant correlation between CatK and LAD (*r* = 0.3, *P* < 0.01). However, there were no correlations between the levels of CatK and the patients’ ages (*r* = 0.04, *P* > 0.05) or BMI (*r* = 0.03, *P* > 0.05).

### Independence of predictors of CHF

The results of the logistic regression analysis for CHF are shown in Table 3 and S3 Table. In the single logistic regression analysis, age, gender, hypertension, LVDd, LAD, and CatK were significantly associated with CHF. However, BMI, diabetes mellitus, hs-CRP, and troponin I were not associated with CHF. The multiple logistic regression analysis using age, gender, hypertension, LAD, LVDd, and CatK revealed that hypertension (odds ratio [OR] 4.11; 95% confidence interval [CI] 1.08–15.69; *P* < 0.05), LAD (OR 1.13; 95%CI 1.01–1.25; *P* < 0.05), LVDd (OR 1.20; 95%CI, 1.05–1.37; *P* < 0.01), and the CatK (OR 0.90; 95%CI 0.84–0.95; *P* < 0.01) levels were significantly correlated with CHF (Table 4 and S4 Table).

**Table 3:** Logistic regression analysis of the associations of various parameters with CHF.

	Single		
	OR Estimate	95% CI	*P*-value
Age (yrs)	1.05	1.02–1.08	<0.01
Gender	0.38	0.18–0.80	<0.05
BMI (kg/m^2^)	0.97	0.89–1.07	0.60
Diabetes mellitus (%)	0.76	0.33–1.75	0.52
Hypertension (%)	3.91	1.83–8.35	<0.001
LAD (mm)	0.94	0.90–0.99	<0.05
LVDd (mm)	0.86	0.82–0.91	<0.0001
hs-CRP (mg/dL)	1.01	0.98–1.03	0.59
Troponin I (pg/mL)	0.98	0.93–1.04	0.50
CatK (ng/mL)	0.94	0.91–0.97	<0.001

The odds ratio (OR) values corresponding to 1 SD increase in each measure of the indicated parameters were estimated. Abbreviations are as in Table 1. CI = confidence interval.

**Table 4:** Independent predictors of CHF according to the multivariable logistic regression analysis.

	OR Estimate	95%CI	*P*-value
Age	0.06	0.05–1.04	> 0.05
Gender	0.22	0.97–1.08	> 0.05
Hypertension	4.11	1.08–15.69	< 0.05
LAD	1.13	1.01–1.25	< 0.05
LVDd	1.20	1.05–1.37	< 0.01
CatK	0.90	0.84–0.95	< 0.01

Abbreviations are as in Table 1.

## Discussion

Several studies obtained evidence that CatK activity controls cardiac remodeling and dysfunction in animals [7,8,23], leading us to raise the possibility that CatK plays a critical role in cardiac disease with heart failure. Few clinical studies reported that cardiac tissues from hypertensive heart failure had increased levels of CatK gene and protein [18]. The findings of the present study provide additional evidence to uphold the possible participation of CatK in cardiac remodeling and dysfunction in patients with CHF.

Recent experimental investigations using transgenic mouse models of CatK deletion have revealed that increased CatK activity contributes to cardiac hypertrophy and cardiac fibrosis with heart failure [3,7,20,22]. To the best of our knowledge, the present study is the first to show that patients with worse LVEF (< 40%) had higher serum CatK levels than did the patients with better LVEF (≥ 40%). Our results also show that CatK was inversely correlated with LVEF. Moreover, we observed that CatK was positively correlated with LVDs and LVDd as well as LAD. The multivariable logistic regression analysis clearly demonstrated that the levels of circulating CatK were an independent predictor of CHF. Past results provide evidence to suggest increased serum levels of the endogenous cathepsin inhibitor cystatin C in association with HF [27–30]. Coupled with a single recent work showing that increased circulating CatK levels were closely linked to the presence of atrial fibrillation and to enhanced levels of collagen degradation products [4], our present findings indicate that the presence of elevated serum levels of CatK can serve as a novel biomarker of CHF and that the monitoring of circulating CatK can be used as a noninvasive way of showing the mechanisms of cardiac remodeling and dysfunction in CHF.

The cysteine protease CatK is one of the most potent mammalian collagenases in cultured cardiovascular cells and its related inflammatory cells including macrophages [7,18,19]. We have previously shown that CatK was expressed in cultured rat neonatal cardiomyocytes and cardiac fibroblasts [19]. Immunostaing analysis also showed that the CatK protein expression was markedly upregulated in the cardiomyocytes of rat myocardium with hypertensive heart failure [19]. Furthermore, in human study, the failing myocardium of patients with hypertensive heart failure had increased levels of CatK mRNA and protein. There is accumulating evidence that CatK degrades extracellular matrix proteins including elastin as well as collagens laminin, and fibronectin [3–5,11,31,32]. Recently, Hua and colleague demonstrated that CatK silencing mitigates diet- or pressure overload-induced cardiac hypertrophy and cardiac fibrosis in animal models [7,23]. Previous study reported that the failing myocardium of patients with hypertrophic and dilated cardiomyopathies had enhanced CatK and CatL compared to control subjects [3]. In 2010, Xie and colleagues revealed that, *in vivo*, myocardial cystatin C was enhanced in hypoxic injury-induced failing myocardium of mice, and this enhance was associated with accumulations of fibronectin and collagen [33]. On the other hand, it is well known that matrix metalloproteinase can degrade all of cardiovascular extracellular matrix proteins and activate several cathepsins in angiogenesis and cardiovascular disease [34–36]. A growing evidence suggests relationships between circulating extracellular matrix metalloproteinase and/or the endogenous tissue inhibitors of matrix metalloproteinase and CHF and the related clinical events in humans [37,38]. Thus, we proposed that CatK might participate in cardiac remodeling and dysfunction by mediating extracellular matrix protein metabolism in cooperation with other proteases, such as serine proteases and matrix metalloproteinases.

There were several study limitations. First, the small sample size of CHF patients limited the power to prove relationships and differences or to conduct the analysis of the lowLVEF and highLVEF groups. On other hand, this study was not designed to determine the difference of circulating CatK in patients with CHF and control subjects. Second, as a serum marker, CatK is not heart-specific. It is difficult to separate CatK as a marker in different arteries and tissues (myocardium, valvular, bone, fat, etc.). Third, we excluded patients with congenital heart disease, end-stage renal disease with maintenance hemodialysis, primary valvular disease, pericarditis, hypertrophic cardiomyopathy, or secondary cardiac muscle disease. It is unclear how their exclusion or inclusion would influence our current findings.

In summary, present findings suggested that the evaluation of peripheral blood CatK may provide a noninvasive way of showing and monitoring both the mechanism and extent of cardiac remodeling and dysfunction in CHF patients. However, further investigations and prospective clinical trials that elucidate the exact role of CatK-related proteolysis in CHF and the importance and value of screening proteolysis and extracellular matrix degradation in clinical settings will be required to conduct near future.

## Supporting Information

S1 Fig Regression line coefficient calculation formula. (PDF)

S1 Table The numbers of II, III, and VI NYHA functional class. (PDF)

S2 Table The umbers of Clinical histories and medication treatments. (PDF)

S3 Table Logistic regression analysis of the associations of various parameters with CHF (raw data). (PDF)

S4 Table Independent predictors of CHF according to the multivariable logistic regression analysis (raw data). (PDF)
