# Supplementary figures and images for: Increased Circulating Cathepsin K in Patients with Chronic Heart Failure
Source: PLoS One. 2015 Aug 24;10(8):e0136093. doi: 10.1371/journal.pone.0136093 (PMC4547812; doi:10.1371/journal.pone.0136093)

S1\_Fig.

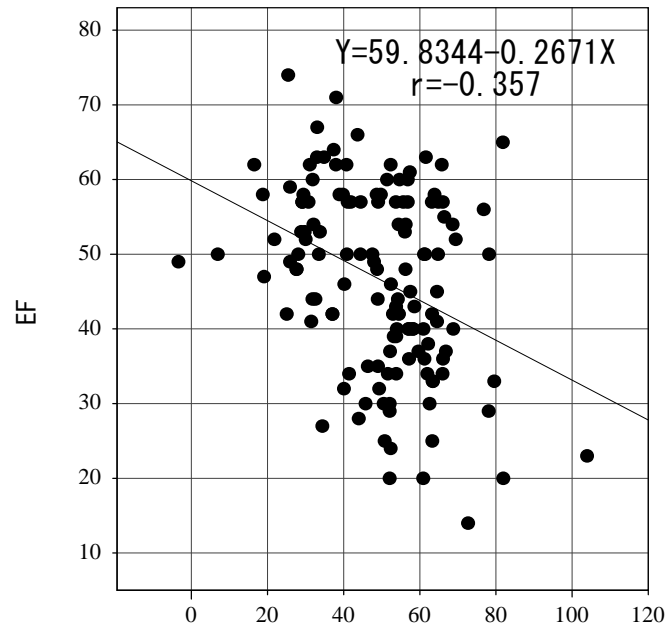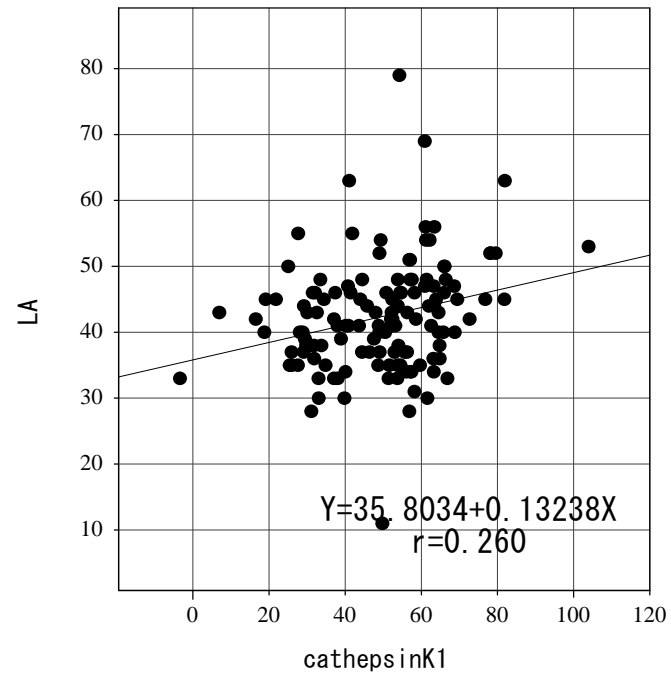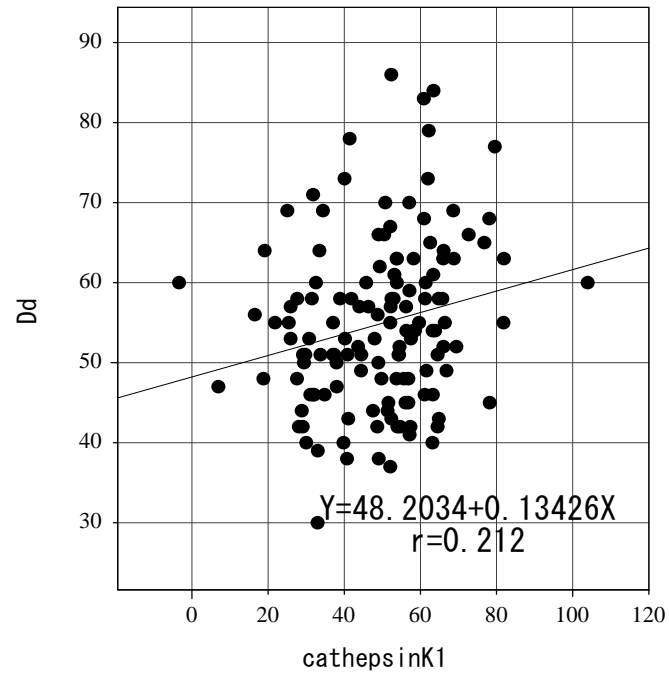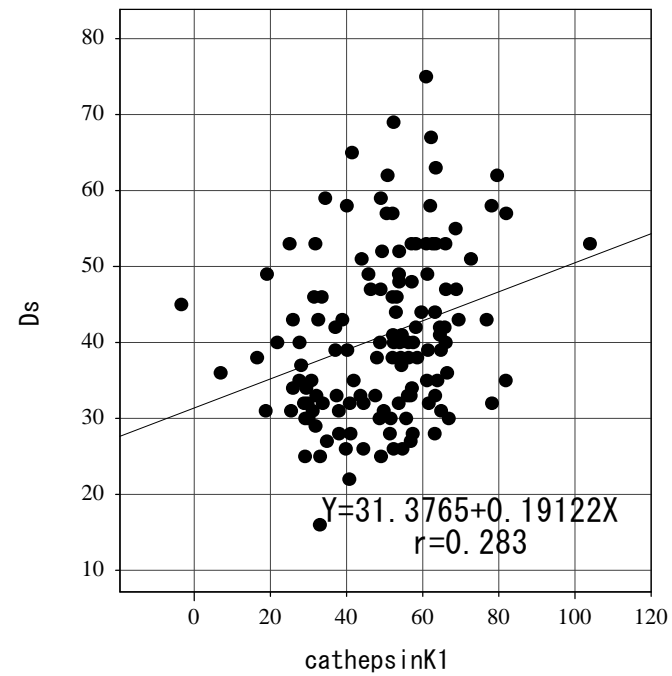

Supplement: S1 Fig — (PDF) [file pone.0136093.s001.pdf]
