# Supplementary material for: Increased Circulating Cathepsin K in Patients with Chronic Heart Failure
Source: PLoS One. 2015 Aug 24;10(8):e0136093. doi: 10.1371/journal.pone.0136093 (PMC4547812; doi:10.1371/journal.pone.0136093)
Supplement: S1 Table — (PDF) [file pone.0136093.s002.pdf]

S1_Table. Patients' characteristics

Age, yrs	66.5 ± 12.9
Female, %	62/134
Body mass index, kg/m^2	23.3 ± 3.1
**NYHA functional class (n)**	
II	17
III	37
VI	70
**Echocardiography**	
LAD, mm	42.3 ± 8.4
IVST, mm	9.3 ± 1.2
LVPWT, mm	12.9 ± 2.0
LVDd, %	55.9 ± 14.5
LVSd, %	48.7 ± 11.9
LVEF, %	41.9 ± 8.3
CI, %	2.8 ± 1.1
**Blood Examination**	
Na^+, mmol/L	139.9 ± 3.7
LDL, mg/dL	90.7 ± 31.0
HDL, mg/dL	45.5 ± 16.2
Hemoglobin A1c, %	5.9 ± 1.4
Creatinine, mmol/L	75.0 ± 32.2
hs-CRP, mg/dL	4.8 ± 7.2
NTproBNP (pg/mL)	4024 ± 4026
Troponin I (pg/mL)	1.5 ± 4.6
CatK, ng/mL	51.6 ± 16.6

Values are expressed as mean ± SD or number (%).

LAD, left atrial dia.; LV, left ventricular; IVST, interventricular septal thickness; LVPWT, LV posterior wall thickness; LVDd, left ventricular end-diastolic dimension; LVSd; LV end-systolic dimension; CI, cardiac index; LDL, low-density lipoprotein; HDL, high-density lipoprotein; hs-CRP, high-sensitivity C-reactivity protein; NTproBNP, N-terminal pro-brain natriuretic peptide; ACEI, angiotensin-converting enzyme inhibitor; ARB, angiotensin type 1 receptor blocker.
