# Supplementary material for: Increased Circulating Cathepsin K in Patients with Chronic Heart Failure
Source: PLoS One. 2015 Aug 24;10(8):e0136093. doi: 10.1371/journal.pone.0136093 (PMC4547812; doi:10.1371/journal.pone.0136093)
Supplement: S2 Table — (PDF) [file pone.0136093.s003.pdf]

S2_Table. Comparison of each parameter between the lowLVEF and highLVEF groups

	**lowLVEF (n=44)**	**highLVEF (n=90)**	***P*-value**
Age, yrs	63.1 ± 13.2	69.8 ± 10.9	0.09
Female, n	34.1	57.8	0.01
Body mass index, kg/m^2	23.6 ± 3.6	22.9 ± 4.0	0.60
**Clinical histories**			
Hypertension, n	17	28	0.12
Diabetes mellitus, n	12	20	0.52
Current smokers, n	15	29	0.83
Previous myocardial infarction, n	16	31	0.86
Previous angioplasty, n	3	15	0.019
Previous bypass surgery, n	0	0	0
Previous cerebrovascular disease, n	1	16	0.011
**Echocardiography**			
LAD, mm	44.9 ± 8.0	41.0 ± 8.3	0.000
IVST, mm	8.9 ± 1.3	9.6 ± 1.2	0.12
LVPWT, mm	11.3 ± 1.9	13.7 ± 1.6	0.08
LVDd, %	62.8 ± 11.1	50.9 ± 7.5	0.000
LVSd, %	55.4 ± 10.0	43.9 ± 3.6	0.000
LVEF, %	32.3 ± 6.5	46.6 ± 3.8	0.021
CI, %	2.6 ± 1.2	2.9 ± 0.7	0.33
**Blood Examination**			
Na^+, mmol/L	139.8 ± 4.4	140.0 ± 3.4	0.74
LDL, mg/dL	90.5 ± 32.5	91.3 ± 28.1	0.81
HDL, mg/dL	44.9 ± 15.4	46.7 ± 17.9	0.77
Hemoglobin, g/dL	12.9 ± 2.2	14.6 ± 2.3	0.09
Serum albumin, g/mL	39.9 ± 5.1	40.5 ± 4.1	0.51
Hemoglobin A1c, %	6.1 ± 1.4	5.8 ± 0.4	0.38
Creatinine, mmol/L	77.4 ± 24.3	73.9 ± 35.4	0.56
hs-CRP, mg/dL	4.3 ± 4.1	5.4 ± 8.4	0.39
NTproBNP (pg/mL)	4269 ± 3757	3599 ± 4225	0.68
Troponin I (pg/mL)	2.1 ± 7.6	1.5 ± 2.3	0.21
CatK, ng/mL	58.4 ± 12.2	44.7 ± 16.4	0.000
**Medications**			
ACEIs, n	17	34	0.92
ARBs, n	21	31	0.14

β-blockers, n	33	64	0.64
Statins, n	28	58	0.64
MR antagonists, n	34	51	0.02
Diuretics, n	31	50	0.020
Digoxin, n	12	14	0.013
Insulin, n	5	16	0.19

Values are expressed as mean ± SD or number (%).

LDL, low-density lipoprotein; HDL, high-density lipoprotein; hs-CRP, high-sensitivity C-reactivity protein; NTproBNP, N-terminal Pro-brain Natriuretic Peptide; ACEI, angiotensin-converting enzyme inhibitor; ARB, angiotensin type 1 receptor blocker; MR, mineralocorticoid receptor.
