# Supplementary material for: Increased Circulating Cathepsin K in Patients with Chronic Heart Failure
Source: PLoS One. 2015 Aug 24;10(8):e0136093. doi: 10.1371/journal.pone.0136093 (PMC4547812; doi:10.1371/journal.pone.0136093)
Supplement: S3 Table — (PDF) [file pone.0136093.s004.pdf]

S3_Table.

Association with CHF

	Single		
	Odds Ratio Estimate	95% CI	*P* value
Age(year)	1.05	1.02-1.08	0.0037
gender	0.38	0.18-0.80	0.0111
BMI (kg/m^2)	0.97	0.89-1.07	0.60
Diabetes mellitus (%)	0.76	0.33-1.75	0.52
Hypertension (%)	3.91	1.83-8.35	0.0004
LAD (mm)	0.94	0.90-0.99	0.0155
LVDd (mm)	0.86	0.82-0.91	0.0000
hs-CRP	1.01	0.98-1.03	0.59
Troponin I (pg/mL)	0.98	0.93-1.04	0.50
CatK	0.94	0.91-0.97	0.0000

The odds ratios corresponding to 1 SD increase in each measure of the indicated parameters were estimated. Abbreviations are as in Table 1. CI = confidence interval.
