# Supplementary material for: Increased Circulating Cathepsin K in Patients with Chronic Heart Failure
Source: PLoS One. 2015 Aug 24;10(8):e0136093. doi: 10.1371/journal.pone.0136093 (PMC4547812; doi:10.1371/journal.pone.0136093)
Supplement: S4 Table — (PDF) [file pone.0136093.s005.pdf]

S4_Table. Independent predictors of CHF according to the multivariable logistic regression analysis

	**OR Estimate**	**95%CI**	***P*-value**
Age	0.06	0.05–1.04	0.18
Gender	0.22	0.97–1.08	0.29
Hypertension	4.11	1.08–15.69	0.0013
LAD	1.13	1.01–1.25	0.010
LVDd	1.20	1.05–1.37	0.000
CatK	0.90	0.84–0.95	0.000

Abbreviations are as in Table 1.
